# Enriched Environment Effects on Myelination of the Central Nervous System: Role of Glial Cells

**DOI:** 10.1155/2022/5766993

**Published:** 2022-04-14

**Authors:** Zhen-Kun Gao, Xin-Ya Shen, Yu Han, Yi-Sha Guo, Mei Yuan, Xia Bi

**Affiliations:** ^1^Shanghai University of Traditional Chinese Medicine, Shanghai 201203, China; ^2^Shanghai University of Sport, Shanghai 200438, China; ^3^Department of Rehabilitation Medicine, Shanghai University of Medicine & Health Sciences Affiliated Zhoupu Hospital, Shanghai 201318, China

## Abstract

Myelination is regulated by various glial cells in the central nervous system (CNS), including oligodendrocytes (OLs), microglia, and astrocytes. Myelination of the CNS requires the generation of functionally mature OLs from OPCs. OLs are the myelin-forming cells in the CNS. Microglia play both beneficial and detrimental roles during myelin damage and repair. Astrocyte is responsible for myelin formation and regeneration by direct interaction with oligodendrocyte lineage cells. These glial cells are influenced by experience-dependent activities such as environmental enrichment (EE). To date, there are few studies that have investigated the association between EE and glial cells. EE with a complex combination of sensorimotor, cognitive, and social stimulation has a significant effect on cognitive impairment and brain plasticity. Hence, one mechanism through EE improving cognitive function may rely on the mutual effect of EE and glial cells. The purpose of this paper is to review recent research into the efficacy of EE for myelination and glial cells at cellular and molecular levels and offers critical insights for future research directions of EE and the treatment of EE in cognitive impairment disease.

## 1. Introduction

Even in the absence of cognitive disease, experience-induced changes in the CNS are commonly associated with myelination. Recent research has suggested that sensory enrichment increased OL integration and myelination in adult mouse somatosensory cortex by accelerating information transfer in circuits and strengthening the ability of axons to sustain activity by providing additional metabolic support [1]. Interestingly, early social isolation negatively regulates prefrontal cortex myelination through neuregulin-1/ErbB3 signaling [2]. Moreover, monocular deprivation changes the length of parvalbumin-expressing interneuron myelin segments in adult mouse visual cortex [3]. Over the decades, a considerable amount of literature has been published to figure out the potential molecular and cellular mechanism of myelination. For example, Piao et al. found that CD44 is an important molecule for enzymoglycan-induced migration of OPCs to focal inflammatory demyelinating lesions, and it may play an important role in OPC repair in multiple sclerosis (MS) [4].

To this end, consistent efforts have been directed toward developing nonpharmacological treatment strategies, such as cognitive behavioral therapy, physiotherapy, lifestyle modifications, and neuromodulation. The evidence presented thus far supports the idea that early and continuous environmental enrichment improves white matter repair in perinatal infants after brain injury by promoting oligodendrocyte maturation, myelination, and cognitive function [5]. In the search for better strategies to promote myelination, the enriched environment (EE) may signify superiority over conventional exercise therapy. There are cases that also demonstrate that EE has extensive and profound effects on rodents from cells and molecules to behavior and mental. For example, EE can upregulate the levels of hypothalamic brain-derived neurotrophic factor (BDNF) and hippocampal vascular endothelial growth factor (VEGF) in the rodent brain [6, 7], which play essential roles in neurogenesis and cognitive function. EE also significantly decreased the frequency and duration of seizures in epileptic rats [8] and depressive-like behaviors in mice models of depression [9].

Cognitive processes such as learning require CNS plasticity throughout life, and several studies have reported the role of neuronal, in particular, synaptic plasticity as a means of altering circuit function. An increasing body of evidence suggests that myelin may also play a vital role in circuit plasticity, and that myelin may be an adaptable structure which could be altered to regulate experience and learning. It was then raised the question that whether myelination of animals might be affected by EE paradigm as well? Recent cases reported that EE could influence myelination. For example, EE could promote remyelination and some ongoing myelination in the white matter of aging rats [10]. EE group exhibited increased myelination, preserved normal protein and mRNA levels of nerve growth factor (NGF), TrkA, PI3K, AKT, ERK, cAMP response element-binding protein (CREB), and myelin basic protein (MBP) in the forebrain regions of rats exposed to chronic immobilization stress [11]. Therefore, EE may open a novel direction in myelination. We need to further discuss the influence of EE paradigm on myelination and glial cells.

## 2. An Overview of Enriched Environment and Future Outlook

From birth through old age, sensory, cognitive, and motor stimuli subtly affect mammalian brain function through interaction with the surrounding environment. However, most animal models that have studied brain function and dysfunction for decades have only involved studying animals in a (standard) housing condition, which provides few opportunities for sensory, cognitive, or motor stimulation. More than seventy years ago, important studies in the animal housing field were initiated by Donald Hebb, who occasionally observed that rats were allowed to run around freely in his house, were better problem learners, and had better memory than rats reared in the laboratory cages [12]. Since then, later studies showed that EE shapes the brain structure and thereby brain function [13, 14].

**Figure 1:**
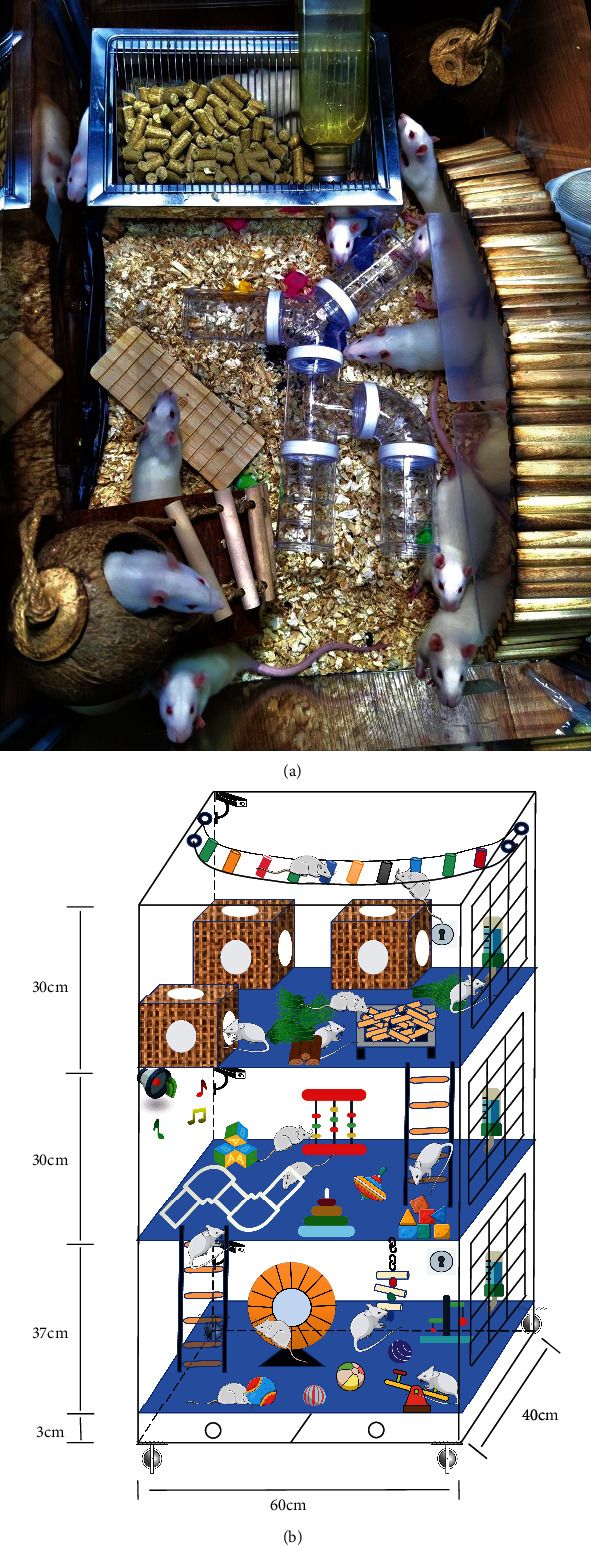
(a) A classic rodent's enriched environment paradigm. There is a larger space in the EE cages to place more animals (8–14 per cage) and various toys, shelters, and other objects to increase sensorimotor stimulation, and the types and positions of objects in the cage are regularly changed to encourage spontaneous exploration. (b) An improved rodent's enriched environment paradigm. The cage is divided into three layers according to the mechanistic routes of EE. In the first layer, voluntary running wheels, climbing ropes, balls, and other objects are placed in the room to enhance locomotion and physical activity. In the second layer, various colored building blocks, toys, and music are used to increase sensory stimuli. In the third layer, shelters, nesting materials, and climbing ladders are placed to increase social interaction and exploratory activities. The objects in EE cage are in line with rodents' habits and nonstressful. Each layer is equipped with a monitor to record the animal's behavior track, and the running wheel is equipped with a sensor to detect the animal's movement. The ladder connecting each floor is easy to pass.

Mark Rosenzweig, one of the pioneers of environmental enrichment studies, defined EE as “a combination of complex inanimate and social stimulation” [15]. In an enriched housing condition, animals, normally rats or mice, are housed in a large cage (8–14 per cage), which has food, colored and textured toys, shelters, running wheels, nesting materials, and so forth, which are replaced and rearranged frequently [16]. Cages used for EE are generally larger than standard cages to make room for more rats and complex and diverse objects (Figure 1(a)). Various animal toys, such as nesting materials and exploratory plumbing, are specifically targeted at the rodent's habits and behavior (digging and climbing) [17]. Animals living in enriched conditions not only are encouraged to exercise and social communicate spontaneously but there also are many opportunities of sensory and cognitive training, compared with an impoverished environment (IE) or standard environment (SE) [18]. There is a case that also supports that EE improves motor, sensory, social, and/or cognitive activities in a voluntary or psychologically stress-free manner [19]. EE has significant effects on a wide range of scales on brain and behavior. One key aspect seems to be explained by the complexity and novelty of EE paradigm [16], the objects in EE cages vary in shape, size, texture, smell, function, and color, and periodically changing the type of objects and the position of objects in the cages. EE cages have greater space to wander, shelters to rest, and novelty objects to explore. Another key aspect seems to be explained by the voluntariness and nonobligatory of EE paradigm. Compared with other involuntary stimulation training (e.g., electroshock therapy and treadmill running), EE can fully improve the initiative of experimental animals to participate in the training with and without psychological pressure. For example, in adult rats with Huntington's disease after embryonic striatal transplantation, EE significantly increased striatal brain-derived neurotrophic factor (BDNF) levels and transplanted neurons showed greater spinal density and larger cell volume, but the forcibly active animals had lower levels of BDNF and fewer graft spines [20]. Compulsive and high-intensity physical activity may show negative effects. Compared with combined exercise training (EE stimuli + aerobic resistance training), the adolescent rats of EE group (EE stimuli + voluntary physical activity) showed reduced levels of hippocampal corticosterone [21]. And corticosterone dysregulations are associated with cognitive impairment and depression, which strongly inhibit adult neurogenesis [22].

Although EE paradigm has made great progress in rodent research in the past few decades, it poses new challenges and problems for this research. EE cages can be filled with voluntary running wheels, a variety of animal toys, and more animals to increase sensory stimulation (Figure 1(a)). However, such as some of the disease, model animals (for example, depressive disorder) are unwilling to move and therefore unable to interact with the objects in the EE cage. We cannot determine if EE paradigm plays a role in the treatment, how much of a role it plays in the treatment process, and which components of EE are more effective in the treatment. Another similar concern is to reduce or eliminate the impact of gender differences and territorial and dominance behavior on animal behavior. Rodents also establish complex social hierarchies that can affect the levels of major urinary proteins (MUPs) [23], and rats of different genders perform differently in behavioral tests [24, 25]. Thus, we design an improved rodent's EE cage for those problems (Figure 1(b)).

Further research on EE paradigm should be undertaken to investigate the continuous personalized behavior caused by environmental change and its potential neurobiological and molecular mechanisms. As Kempermann [26] described, in most EE paradigms, the experimenter seldom interferes with the individual behavior, exploration, and activity of animals. However, the differences between these individual behaviors may also have a profound impact on behavior and the brain. Interestingly, the evidences presented thus far support the idea that EE can indeed increase differences between groups [27, 28]. With the rise of personalized precision medicine, EE paradigm may be used as a model of individualized biology and/or disease to help us better to dissect neurobiological mechanisms. In addition, Nithianantharajah and Hannan [16] summarized the mechanistic routes of EE to the brain as follows: sensory, cognitive, motor, and social stimulation. We could quantify the different components of EE paradigm and analyze their contribution to brain plasticity and disease treatment. What is worth mentioning, this case study confirms that both voluntary wheel running and EE increased the number of new cells and neurogenesis in the adult mouse dentate gyrus [29]. This can better help experimenters to design and adjust EE paradigms for diverse animal and disease models (such as demyelinating models, adding more sensory stimulation to promote the proliferation, and differentiation of OL cells), to improve the therapeutic effects.

## 3. Effect of Enriched Environment on Myelination

**Figure 2:**
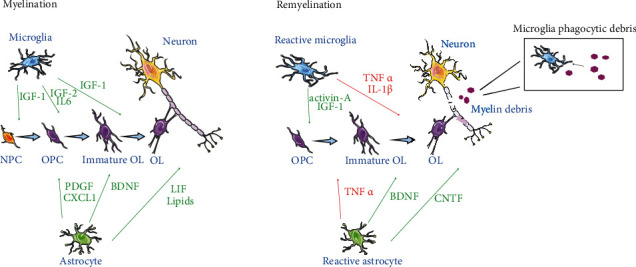
Myelination and remyelination of glial cells in the CNS. In the process of myelination, NPC matures into OPC, OPC proliferates and differentiates into immature OL, which then forms myelin sheaths that wrap axons, and astrocyte and microglia release various molecules to control the development of OL and myelination. When the myelin sheath is damaged and repaired, OPC proliferates and differentiates to supplement OL, astrocytes and microglia enter an activated state, and both exhibit dual activities, inducing harmful or beneficial effects. In addition, microglia can swallow fragments of the myelin sheath. The green arrows indicate molecules exhibiting positive activity on oligodendrocyte lineage and myelin production, whereas the red arrows indicate molecules displaying inhibiting activity on oligodendrocyte lineage production.

Many literatures have shown that the brain responds to experience-induced activities [30–33] and environmental changes [34, 35]. Changes in myelin sheaths, neurons, and synapses can be observed both in animal and cell culture models. Hence, whether we can use the effect of increased sensory and motor stimulation to promote myelination? This case shows that exposure of 1-month-old solitary mice to rich feeding conditions for 8 weeks decreased cellular apoptosis, synaptic protein loss, myelination defect, and microglial activation in the hippocampus, but not the medial prefrontal cortex (mPFC) of mice housed singly [36]. Different from dead neurons, the plasticity of myelin and oligodendrocyte lineage cells can be alleviated or restored after brain injury, thus, the cognitive and mnemonic impairments associated with myelin would be repaired. In contrast, the mechanisms by which EE influences glial cell production and myelination remain relatively unknown. Myelination and remyelination of axons in the CNS are a concerted effort between various cell types, resulting in significant cross-talk and communication among cells (Figure 2). Myelination is guided primarily by the interaction of oligodendrocyte lineage cells with other glial cells: microglia and astrocyte [37]. Moreover, the interaction of oligodendrocyte lineage cells with neurons cannot be forgotten, even if this theme is not the focus of this review. In fact, neurons are the substrate of myelination, and it is fully established that neuronal dysfunctions affect myelination and vice versa [38]. To explore the goals of EE, the first step is to figure out what happens to glial cells during CNS myelination.

**Figure 3:**
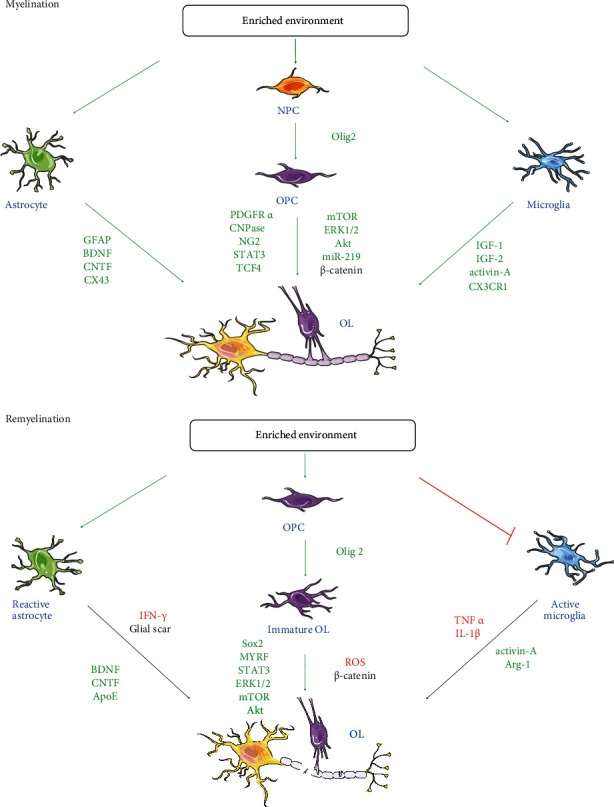
An overview of the effect of enriched environment on glial cells in myelination and remyelination. Please refer to the text for details. The green arrows indicate molecules exhibiting positive activity on oligodendrocyte lineage and myelin production, the red arrows and blocking symbols indicate molecules displaying inhibiting activity on oligodendrocyte lineage production whereas the black arrows indicate molecules exhibiting dual activity on oligodendrocyte lineage and myelin production.

Here, we confine our discussion to preclinical animal studies, particularly in rats and mice, where most studies exploring the effects of EE on myelination and glial cells have been conducted (Figure 3).

### 3.1. EE and Oligodendrocyte Lineage Cell

Oligodendrocytes are one of the main types of glial cells in the central nervous system except for microglia and astrocytes. A single mature OL product about 100 myelin sheaths [39], oligodendrocytes are indispensable to myelin formation in the developing stage and important for remyelination after injury. In the process of myelin development or remyelination, oligodendrocytes produce a large amount of membrane proteins and lipids to form a myelin sheath to wrap axons. Therefore, the myelin sheath is an extension of oligodendrocytes, which tightly wraps nerve axons in a concentric manner.

Although oligodendrocyte precursor cells (OPCs) account for 5-8% of all cells in the CNS [40], it has commonly been assumed that OPC is generated from neural progenitor cell (NPC) and gives rise to mature OL in the CNS [41]. The differentiation of OPCs into mature and myelin-producing OLs is a complex process that is tightly regulated by specific transcription factors, extracellular signals, epigenetic modifications, and signaling pathways. PDGFR*α* is a marker of OPC and plays an important role in myelination of the central nervous system. Therefore, experiments in transgenic mice show that PDGF*α* drives OPCS to divide, and overexpression of PDGF*α* can induce OPC overproliferation and oligodendrocyte overproduction [42]. 4-week EE enhanced BrdU positive cells in all cortical areas of adult spontaneously hypertensive stroke rats, and some BrdU positive cells were identified as PDGFR*α* positive OPC [43]. The NPCs, which come from the subventricular zone (SVZ), have the potential to generate OPCs in the developing CNS [41]. EE not only promotes the proliferation and differentiation of OPCs but also increases the number of NPCs. Pusic and Kraig [44] demonstrated that stimulating OPC differentiation via exosomes derived from environmentally enriched animals is unlikely to deplete progenitors, as EE itself promotes the proliferation of NPCs. Adult spontaneously hypertensive rats were treated with cortical infarcts, and then allowed to survive for 5 weeks in EE and standard environment, EE increased the level of NPC (Ki67 and phosphorylated histone H3 were marked cell proliferation, Sox-2 were marked neural stem/progenitor cells) in the SVZ [45]. OPC generates new oligodendrocytes to replace lost cells after injury and plays an important role in remyelination. In the demyelination response, the increased expression of Sox2 can help adult OPCS transform into an activated state to promote the proliferation and differentiation of OPC [46]. There is case support that 3 weeks of EE + exercise therapy significantly increased hippocampal Sox2 expression and spatial memory function of rat [47]. In Sox2-deficient postoperative cognitive dysfunction (POCD) mice, decreased Sox2-positive cells in the hippocampus can be observed, but EE attenuates POCD by maintaining sox2-positive cells in the hippocampus [48].

After the start of myelination, the preoligodendrocytes combine with the target axon and begin to form filamentous myelin branches. At this stage of differentiation, preoligodendrocytes are characterized by the expression of CNPase [49]. Interestingly, Zhao et al. [50, 51] found EE increases the total number of CNPase positive cells in middle-aged rat corpus callosum and in the hippocampal CA1 and dentate gyrus (DG) of aged rats. And EE and reaching training of the dominant forelimb on day 42 significantly increased the number of OLs in the newly differentiated CNPase+ sensory-motor cortex, and myelin levels in the frontal cortex increased as measured by CNPase expression [52].

Oligodendrocyte transcription factor 2 (OLIG2) is considered as a major and indispensable transcription factor during different stages of OL development, which as a positive factor of OL differentiation and regeneration in adult life [53]. The regulation of the functional specificity and activity of a Smarca4/Brg1-dependent chromatin-remodeling complex by Olig2 is critical to initiate the transcriptional program that promotes OLs differentiation and myelination in the CNS [54]. Correspondingly, EE increased the number of bromodeoxyuridine (BrdU) positive (newborn) cells in the mouse amygdala, and almost all cells expressed the oligodendrocyte precursor marker Olig2 immediately after 1 week of enrichment [55].

NG2 cells have a potential function to proliferate and differentiate into OLs. The combination treatment with Xiaoshuan enteric-coated capsule (XSEC) and EE robustly increased the number of NG2+ cells in the peri-infarct cortex and striatum of MCAO rats by immunofluorescence staining [56]. And the proliferation of NG2 cells increases in response to specific reaching training of the dominant forelimb (RT) and EE in the sensorimotor cortex at day 10 [57]. Oligodendrocyte transcription factor myelin regulatory factor (MYRF) plays an important role in myelin formation during development. It has been proved that after MYRF deletion in OPCs, subsequent remyelination in the spinal cord and corpus callosum is seriously damaged, and the ability of single OPC derived oligodendrocytes to express myelin protein in demyelination reaction is very small [58]. Correspondingly, EE increases MYRF levels in immobilization stress (IS) rats and increases transcription factors required for myelin membrane formation [59]. STAT3 is indispensable in the development of myelination in the central nervous system, and Steelman et al. [60] also proved that activation of oligodendroglia Stat3 promotes oligodendrocyte regeneration and effective remyelination after toxin-induced focal demyelination in the adult brain. EE can promote neurogenesis by increasing the expression of transcriptional activator 3 (p-STAT3) in the ischemic hemisphere of MCAO mice [61]. And the combined application of EE and conditioned reflex sciatic axotomy (SNA) before spinal cord injury (SCI) can significantly improve the p-STAT3 level of dorsal root ganglion (DRG) in SCI mice, to improve axon regeneration [62]. Wedel et al. [63] found that the transcription factor 4 (TCF4) was the preferred heterodimerization partner of the central regulator of OL development, Olig2. Young adult Tcf4 heterozygote knockout mice showed lower neural progenitor cell number. However, EE could enhance the maturation and survival of new neurons and substantially augment the neurogenesis levels of Tcf4 heterozygote knockout mice [64]. Hence, the potential protection mechanism of EE maybe could be explained by the interaction between OLIG2 and TCF4.

Myelination is a highly regulated process that is governed by several signaling pathways.

The wingless and integration site (Wnt) intracellular signaling pathway is a key signaling pathway regulating the development of OL, including OPC proliferation, differentiation, myelination, and remyelination. Interestingly, the Wnt pathway can not only inhibit myelination but also promote myelination [65]. Knockout of *β*-catenin in mice can lead to reduction of myelin gene expression and myelination, and loss-of-of-function analysis of zebrafish embryos showed that inhibition of Wnt/*β*- Catenin signal leads to low myelination [66]. EE can improve spatial learning and memory and the levels of Wnt pathway proteins in rats with vascular dementia [67]. Qu et al. [68] also found EE activated the Wnt/*β*-catenin signaling pathway in the hippocampus of chronic cerebral hypoperfusion (CCH) rats and concluded that EE improved cognitive impairment could be the protection of the blood-brain barrier (BBB). However, a number of studies also have examined the inhibitory effect of Wnt signaling on myelination. In mice activated by Wnt/beta-catenin signal, it can be observed that mature oligodendrocytes, myelin proteins, and myelinated axons appear to be delayed during development. The underlying mechanism is the activation of Wnt pathway through catenin to inhibit the maturation of oligodendrocytes [69]. Correspondingly, it has been found that EE can significantly enhance remyelination and recovery of exercise capacity in a mouse model of demyelination induced by cupoproxol. The underlying mechanism may be that EE inhibits the Wnt signaling pathway during remyelination by increasing the expression of HDAC1/2 [70]. A large number of in vitro and in vivo studies have shown that the AKT/mTOR signaling pathway plays an important role in promoting myelination in the central nervous system. Constitutively, active Akt can enhance the myelination of the central nervous system of mice, and as the mouse ages, the increase of myelin sheath continues, leading to the enlargement of the optic nerve and white matter area [71]. EE plays a positive role in myelination by reversing the decrease of Akt and MBP levels in the forebrain area of chronic fixed stress rats [11]. mTOR is a well-known Akt substrate. By knocking out mTOR in the central nervous system of mice, it was observed that the expression of myelin proteins in the spinal cord, including myelin basic protein, proteolipoprotein, myelin oligodendrocyte glycoprotein, and myelin associated glycoprotein, was delayed; moreover, the low myelination of the spinal cord continued until adulthood, as did the decrease in the number of mature oligodendrocytes [72]. mTOR that was inhibited by rapamycin could reduce myelination in young adult wild-type mice starting at 6 weeks of age [73]. Interestingly, EE protects myelin membrane from oxidative damage caused by chronic stress by upregulating myrf and mTOR mRNA levels in the forebrain of immobilization stress (IS) rats [59]. The extracellular signal-regulated kinase (ERK) pathway can transduce extracellular signals through intracellular signal cascades and control the expression of genes that regulate the proliferation, differentiation, and survival of oligodendrocytes and other important processes [74]. The ERK signaling could active during the rapid growth and thickness of myelin and promote OL differentiation and the initiation of myelination in Erk1/2 KO mice [75]. It is gratifying that EE promotes myelination and increases myelin thickness by improving ERK mRNA levels in the forebrain of rats with chronic fixation stress [11].

OPCS and oligodendrocytes are very vulnerable to oxidative stress due to their poor ability to scavenge peroxides and high iron content in cells. Especially in the process of myelination, oligodendrocytes are vulnerable to cytotoxic by-products produced by high metabolism, such as reactive oxygen species (ROS) and hydrogen peroxide [76]. Interestingly, EE can reduce the oxidative state of the mouse hippocampus and medial temporal cortex and reduce hippocampal cell apoptosis [77]. EE treatment for 21 or 28 days can attenuate the negative impact of unpredictable chronic stress (UCS) on zebrafish behavior and prevent the impact on ROS levels [78].

Recently, more attention has been paid to the exciting therapeutic potential of miRNAs in promoting myelination. For example, the direct targets of miR-219 include genes essential for maintaining OPC proliferation (e.g., Sox6, Lingo 1, and PDGF*α*), and its increase stimulates OPCs to exit from the proliferative [79]. Correspondingly, EE rat serum-derived exosomes are rich in miR-219, which increases OPCs and their differentiation into mature myelin-forming cells by reducing the expression of differentiation inhibitory regulators [44]. And exosomes isolated from various circulating immune cell types in EE rats increased the content of myelin and miR-219 in the slice culture and reduced the level of oxidative stress [80].

The main functions of oligodendrocytes in the central nervous system are myelination during development, adaptive myelination in adulthood, and remyelination after injury, while OPC is mainly used as a reserve pool for the generation of new oligodendrocytes.

On the contrary, some recent examples have shown that OPC may have an inhibitory effect on myelination. For example, in inflammatory demyelinating mice, OPC differentiation was inhibited by effector T cells and IFN *γ*, and the number of OPC was also decreased by IFN *γ*. This case shows that inhibiting OPC-mediated inflammation may improve cell death and facilitate the differentiation of OPC into myelin-producing oligodendrocytes [81].

Although there are similarities between animals and cells in the development and differentiation of OPCS and oligodendrocytes, there are still differences that need to be considered when applying the effects of EE on OLs and OPCs to humans. We have a good understanding of the underlying mechanisms of oligodendrocytes in myelination and remyelination, but the molecular mechanisms of EE's involvement in these processes still need to be further explored.

### 3.2. EE and Microglia

Microglia are required for normal developmental myelination, which can support remyelination, synapse formation, circuit sculpting, myelination, plasticity, and cognition.

In recent decades, the exploration of the molecular mechanism of microglia in myelination has never stopped. Hamilton and Rome [82] supported the first evidence of the role of microglia in myelin development. They found that the coculture of microglia and oligodendrocytes increased the myelin-specific proteins in oligodendrocytes, MBP, and PLP. And the cooperation of OPC-intrinsic GPR17 signaling and microglia led to extensive myelination of regenerated axons in injured optic nerves [83].

In the process of myelination, microglia promote myelin regeneration by phagocytizing myelin fragments and secreting cytokines and growth factors. Microglia can produce insulin-like growth factor-1 (IGF-1), which enhances microglia induces OLs differentiation [84]. The mechanism of oligodendrocyte differentiation induced by microglia may be related to IGF-1. It has been proved that there is a high level of IGF-1 in the medium derived from microglia, and the expression of IGF-1 can be detected in microglia located in the corpus callosum on the 7th day after birth [85]. And it has been documented that IGF-1 knockout mice reduce the survival, differentiation, and maturation of oligodendrocytes [84]. Accordingly, EE increased the hippocampus levels of IGF-1 in Alzheimer's disease (AD) mice [86]. Baroncelli et al. [87] found IGF-1 levels are increased in the visual cortex of EE rats as early as P6, and blocking IGF-1 signaling prevented the deployment of EE effects, indicating IGF-1 is a key factor mediating EE promoting OL differentiation induced by microglia. Microglia-derived IGF-2 can also prevent TNF*α*-induced death of mature oligodendrocytes in vitro [88]. EE can increase the mRNA and protein expression levels of hippocampal IGF-2 in the offspring of prenatal chronically stressed rats [89]. Microglia express activin-A in demyelinating diseases, which is necessary for myelination after development and injury. There is case support that knocking out all activin-A receptor signal components in oligodendrocytes proves that activin-A is a new therapeutic target for myelin production after injury, and the underlying therapeutic mechanism is that those signals form a myelin sheath by regulating oligodendrocyte differentiation and myelin compaction [90]. Although there is no direct evidence, there are reports in the literature that EE can increase the expression of activin-A in the brain of mice with chronic hypoxic-ischemic brain injury [91], and EE can also increase the expression of activin-A in the brain during chronic cerebral ischemia (CCI) injury [92]. The key role of microglia in the phagocytosis of myelin fragments is essential to promote the differentiation and proliferation of OPC into myelin cells [93]. CX3C receptor 1 (CX3CR1) plays an important role in controlling microglia physiology and coordinating the process of microglia phagocytosis of myelin. In CX3CR1 deficient mice demyelinated by copper-zinc strips, the efficiency of microglia in removing myelin fragments is low, which affects the integrity of axons and myelin, thus hindering the normal remyelination process [94]. Interestingly, CX3CR1 deficiency can increase hippocampal plasticity and spatial memory, while weakening EE increases the number and migration of neural progenitor cells in mouse DG [95], and EE and odor enrichment (OE) did not improve the memory functioning or neurogenesis of and had no effect on microglia in CX3CR1(-/-) mice [96]. Those cases indicate that CX3CR1-mediated signals are essential for the normal function of EE to promote OPC proliferation and differentiation through microglia.

As the resident immune cells of the CNS, microglia will be activated when they detect any damage. Activated microglia also release some cytokines (TNF, interleukins), chemokines, complement, cell adhesion glycoproteins (integrins, selectins), and neurotrophic factors to the adjacent axis damage to myelin and oligodendrocytes [97]. Tumor necrosis factor (TNF) is a key component of the inflammatory response under pathological conditions. LPS-activated microglia polarized to a proinflammatory status secrete TNF*α* and interleukin-1*β* (IL-1*β*), which both known to be cytotoxic for OLs [98]. Interestingly, EE housing blocks higher expression levels of TNF-*α* in microglia from depressive-like mice and regulates microglia phenotype and activation state [99]. EE can also inhibit the levels of activated microglia and TNF*α* and IL-1*β* in rats with optic neuritis [100]. However, it has been reported in the literature that in demyelinating mice lacking TNF-*α* and its related receptors, a significant delay in remyelination and repair failure and proliferation of oligodendrocyte progenitors have been observed with the decrease of the cell pool and the decrease of the number of mature oligodendrocytes. The underlying mechanism is that TNF*α* promotes the proliferation and remyelination of oligodendrocyte progenitor cells through its TNFR2 receptor [101]. Correspondingly, blood samples from EE mice showed higher TNF-*α* mRNA concentrations than infected subjects, microglia from a single (DENV1) serotype infected mice maintained in EE showed lower morphological complexity [102], and EE increases the extracellular levels of TNF*α* and prevents some morphological features of microglial inflammation in AD model [103].

Microglia have dual activities in the process of myelin repair, namely, the activation state related to proinflammatory activity (M1) and the polarization state related to OPC recruitment and differentiation related to anti-inflammatory activity (M2). M2 cell polarization is essential for OLs differentiation in the CNS. In vitro experiments, it was found that when remyelination begins, microglia switch from M1 to M2, and in the coculture of M2 microglia and oligodendrocytes, the differentiation of oligodendrocytes in vitro is enhanced, but after M2 cell injury in vivo, the differentiation of oligodendrocytes is weakened [104]. Interestingly, the phenotypic changes of microglia can be affected by EE paradigm. Exposed to EE increased neocortical levels of interleukin-11 (IL-11), which polarized microglia toward an M2a-dominant phenotype [105]. It has been reported that pathological analyses of early social isolation (SI) mice found that EE could decrease myelination defects and microglial activation in the hippocampus [36]. EE can induce anti-inflammatory M2 polarization of microglia through selective expression of Arginase1 gene (Arg-1) in the hippocampus [106], and this case also supports that EE can alleviate and change the depression-like behavior of depression model rats by regulating microglia phenotype, inhibiting proinflammatory factors and promoting anti-inflammatory factors [107]. In abdominal surgery rat models with postoperative cognitive dysfunction (POCD) and neuroinflammation, preoperative environmental enrichment (PEE) attenuated surgery-induced impairment of novel object preference and reversed the proinflammatory phenotype of hippocampal microglia [103]. Except that influence to rodents, EE piglets showed the gene upregulation involved in neuronal activity and synaptic plasticity of the frontal cortex and a relative decrease in microglial activity [108]. Different microglial subpopulations could potentially influence myelination-related processes. Therefore, another possible area of future research would be to investigate the interaction between EE and microglia.

Microglia are essential in the development and repair of oligodendrocytes and myelination. During development, microglia secrete different patterns of molecules to control the development and myelination of oligodendrocytes, leading to subsequent myelination. In addition, under pathological and demyelinating conditions, activated microglia exhibit dual activities, inducing harmful or beneficial effects. The balance of the two seems to be essential for tissue regeneration. At the same time, microglia can swallow fragments of myelin. Interestingly, EE promotes myelination or remyelination by affecting these physiological and pathological processes of microglia. Further exploration of this process will pave the way for EE to become a new treatment method aimed at restoring normal development of myelination and eliminating the barriers to failure of damaged remyelination in demyelinating diseases of the central nervous system.

### 3.3. EE and Astrocyte

Astrocytes are well known to play critical roles in the CNS. In addition to specific functions in synaptic signaling and blood-brain barrier (BBB), astrocytes are essential for myelination and repair [109]. In the mature brain, the highest cholesterol content is myelin. Therefore, myelination requires a large amount of lipids [110]. Correspondingly, an important factor in the difficulty of remyelination is the lack of transcription factors for lipid synthesis. Due to the high demand for lipids during myelination, a substantial fraction of lipids incorporated into OLs is contributed by astrocytes in the vertebrate CNS [111]. In the absence of astrocyte lipid synthesis, oligodendrocytes cannot complete myelination, resulting in hypomyelination and slow conduction fibers.

Glial fibrillary acidic protein (GFAP) is a specific marker of astrocytes. GFAP knockout mice exhibited myelin ultrastructural defects, including unmyelinated axons in the optic nerve, reduced myelin thickness in the spinal cord, and myelin relaxation [112]. Transient ablation of GFAP+ astrocytes in the mouse spinal cord during the first postnatal week reduced the number of mature OLs and inhibited myelin formation, while prolonged ablation resulted in myelin that lacked compaction and structural integrity, ablation of GFAP+ astrocytes in the adult spinal cord resulted in the rapid, local loss of myelin integrity, and regional demyelination [113]. Interestingly, our previous study demonstrated that EE can increase the number of GFAP-positive astrocytes in the hippocampus of rats after ischemic stroke [114]. Rahati et al. [115] documented that EE can reverse the decrease of GFAP positive cells in the prefrontal cortex of schizophrenic rats. EE paradigm significantly elevated astrocytes (GFAP) activity after intracerebral hemorrhage (ICH) [116].

In the process of myelination, astrocyte promotes myelin regeneration by secreting cytokines and growth factors. For example, BDNF is necessarily required for myelination during early postnatal development. The mRNA expression levels of myelin protein PLP and MBP in BDNF knockout heterozygous mice decreased significantly in hippocampus and cortex at P20 [117]. During endogenous recovery from white matter damage, astrocytes accelerate oligodendrogenesis by secreting BDNF both in vivo and in vitro [118]. Interestingly, environmental enrichment is an experimental paradigm for the increase of brain-derived neurotrophic factor (BDNF) gene expression in the hippocampus of rodents. EE increases the expression of BDNF mRNA in the hippocampus of mice through continuous epigenetic modification [119]. And Chen et al. [120] revealed that rats in physical and social enrichment group (PSE) and physical enrichment group (PE) groups showed significantly more proliferated astrocytes and higher expression levels of BDNF in the peri-infarct cortex by double immunofluorescent labeling and western blot analysis, and astrocyte proliferation and BDNF expression were significantly correlated with cognitive function. In our study, we found that EE can promote myelin regeneration by stimulating the expression of BDNF in astrocytes in the hippocampus of rats after ischemic stroke and then improve the cognitive function of rats. Astrocytes also secrete ciliary neurotrophic factor (CNTF), which acts on oligodendrocytes and has strong promyelinating effects [121]. Interestingly, EE increased the relative expression of CNTF mRNA in retinal extracts of mice with retinitis pigmentosa (RP) [122]. Astrocytes become reactive after myelin injury in the central nervous system. Reactive astrocytes release proinflammatory factors. The apolipoprotein E (APOE), predominantly expressed by astrocytes in the CNS, is another important lipid and cholesterol transport molecule as well as an immunomodulator [123]. EE after MCAO caused a significant decrease in APOE levels of ApoE/S100beta (+) reactive astrocytes, and the improved recovery of neurologic function is associated with a marked reduction of APOE levels [124]. Reactive astrocytes also release IFN-*γ*, which plays an important role in experimental autoimmune encephalomyelitis (EAE). Blocking the IFN-*γ* signaling pathway of astrocytes can reduce the expression of central nervous system cytokines and chemokines, thereby preventing demyelination [125]. However, some literature found that EE improves the inflammation of astrocytes by upregulating the serum IFN-*γ* level of Alzheimer's disease mice [126]. This phenotype may be caused by different disease models.

Connexin (Cx) 43 on astrocytes forms gap junctions with Cx32 and Cx47 on oligodendrocytes, thus, transporting molecules from astrocytes to oligodendrocytes. This case has shown that the deletion of Cx47 and CX30 in mice will cause serious myelin defects, including thin myelin sheath and a decrease in the number of oligodendrocytes [127]. Accordingly, this case confirms that EE can increase the expression of Cx43 in the hippocampus of young rats with ischemia and hypoxia [128].

Reactive astrocytes can form a glial scar, which has beneficial effects at one phase of the remyelination process and destructive effects at another phase [129]. The growth inhibitory effect of glial scars has been considered as the main obstacle to regeneration in the previous literature and to a certain extent explains the lack of effective recovery of central nervous system remyelination. Interestingly, exposure to multimodal early-onset stimulation (MEOS) combined with environmental enrichment (EE) after traumatic brain injury (TBI) can reduce central nervous system glial scar formation and improve nerve recovery after traumatic brain injury in rats [130]. This case also supports that EE can increase the number of astrocytes and reduce the volume of glial scars in young and old rats after ischemic stroke [131]. However, many literatures have proved that glial scar tissue may play an important role in the repair of myelin regeneration in the central nervous system. Glial scar is considered as a physical barrier to prevent the spread of inflammation, which can limit the migration of leukocytes to brain-damaged tissues [132]. So, astrocytes have both supportive and detrimental functions in myelination and remyelination. The expression of sigma-1 receptor, which exists in reactive astrocytes and neurons and could enhance cellular transport of biomolecules required for brain repair, is increased in the peri-infarct areas of rats housed in an enriched environment for two weeks after permanent middle cerebral artery occlusion (pMCAO) [133]. The physiology and pathology of astrocytes are responsible for OLs and myelin [109, 111, 113], thus, the quantity and morphological changes of astrocytes could influence myelination during the process of EE treatment.

In summary, most of the experimental evidence reported in this review indicates that EE promotes myelination and repair by affecting astrocytes. During development, astrocytes secrete different patterns of molecules to control the development and myelination of oligodendrocytes. In addition, astrocytes provide a large amount of lipids to the myelin of the central nervous system, which is necessary for the normal production of the myelin sheath. Under pathological and demyelinating conditions, reactive astrocytes exhibit dual activity, inducing harmful or beneficial effects.

Astrocytes have been associated with both beneficial and detrimental functions in myelination. Given the emerging evidence for the critical role of astrocytes in myelination, further understanding of EE effects on astrocytes in myelin development is necessary.

## 4. Conclusion

In this review, we describe the working rationale of the EE paradigm and its impact on rodents and future research directions and explain the impact of EE on myelination from the perspective of glial cells. This literature demonstrates that EE and voluntary exercise can promote oligodendrocyte differentiation and myelination capacity, and correspondingly, social isolation negatively regulates myelination [134]. It is worth mentioning that under the influence of EE, glial cells interact to promote myelination, which has a profound impact on the improvement of cognitive function. Therefore, many literatures explain the mechanism of EE improving cognition function from the effect of EE on glial cells. However, the research in this field is not deep enough. There is still a long way to go to fully understand the therapeutic mechanism of EE promoting myelination from preclinical and clinical experiments. The proliferation and activation of glial cells involved in myelination are not necessarily from external administration or stem cell transplantation. On the contrary, the noninvasive stimulation strategy of EE paradigm can be used to stimulate myelination in the CNS. Compared with other sensory stimulation paradigms (e.g., treadmill exercise and electroacupuncture), the stimulation produced by EE paradigm is efficient and psychologically nonstressful, and the use of experimental pathology and noxious stimulation can be avoided. We firmly believe that the EE paradigm may be a feasible alternative treatment strategy, possibly highly successful for the treatment of cognitive disorders and demyelinating diseases.

## Data Availability

The [DATA TYPE] data used to support the findings of this study are included within the article.
